# A review of protective factors and causal mechanisms that enhance the mental health of Indigenous Circumpolar youth

**DOI:** 10.3402/ijch.v72i0.21775

**Published:** 2013-12-09

**Authors:** Joanna Petrasek MacDonald, James D. Ford, Ashlee Cunsolo Willox, Nancy A. Ross

**Affiliations:** 1 Department of Geography, McGill University, Montreal, Quebec, Canada; 2 Departments of Nursing and Indigenous Studies, Cape Breton University, Sydney, Nova Scotia, Canada

**Keywords:** mental health, resilience, Arctic, Indigenous, young people, Inuit, Sami, Inupiat

## Abstract

**Objectives:**

To review the protective factors and causal mechanisms which promote and enhance Indigenous youth mental health in the Circumpolar North.

**Study design:**

A systematic literature review of peer-reviewed English-language research was conducted to systematically examine the protective factors and causal mechanisms which promote and enhance Indigenous youth mental health in the Circumpolar North.

**Methods:**

This review followed the Preferred Reporting Items for Systematic Reviews and Meta-Analyses (PRISMA) guidelines, with elements of a realist review. From 160 records identified in the initial search of 3 databases, 15 met the inclusion criteria and were retained for full review. Data were extracted using a codebook to organize and synthesize relevant information from the articles.

**Results:**

More than 40 protective factors at the individual, family, and community levels were identified as enhancing Indigenous youth mental health. These included practicing and holding traditional knowledge and skills, the desire to be useful and to contribute meaningfully to one's community, having positive role models, and believing in one's self. Broadly, protective factors at the family and community levels were identified as positively creating and impacting one's social environment, which interacts with factors at the individual level to enhance resilience. An emphasis on the roles of cultural and land-based activities, history, and language, as well as on the importance of social and family supports, also emerged throughout the literature.

More than 40 protective factors at the individual, family, and community levels were identified as enhancing Indigenous youth mental health. These included practicing and holding traditional knowledge and skills, the desire to be useful and to contribute meaningfully to one's community, having positive role models, and believing in one's self. Broadly, protective factors at the family and community levels were identified as positively creating and impacting one's social environment, which interacts with factors at the individual level to enhance resilience. An emphasis on the roles of cultural and land-based activities, history, and language, as well as on the importance of social and family supports, also emerged throughout the literature.

**Conclusions:**

Healthy communities and families foster and support youth who are resilient to mental health challenges and able to adapt and cope with multiple stressors, be they social, economic, or environmental. Creating opportunities and environments where youth can successfully navigate challenges and enhance their resilience can in turn contribute to fostering healthy Circumpolar communities. Looking at the role of new social media in the way youth communicate and interact is one way of understanding how to create such opportunities. Youth perspectives of mental health programmes are crucial to developing appropriate mental health support and meaningful engagement of youth can inform locally appropriate and culturally relevant mental health resources, programmes and community resilience strategies.

Indigenous youth in communities across the Circumpolar North experience significant health disparities and poorer mental health, however measured, than non-Indigenous youth (1–9). Inuit and Inupiat youth suicide, for instance, is at “epidemic” levels, with communities experiencing some of the highest suicide rates globally (5, 10–14). Between 1999 and 2003, Inuit communities in Nunavik, Northern Quebec, experienced suicide rates 15 times higher than that of the Canadian average (181/100,000, while the region of Nunatsiavut had the highest suicide rate in Canada (239/100,000) (15). Suicide rates for 15 to 24 year-old Alaska Native males is 14 times the national rate (16) and, in northwest Alaska, suicide is the leading cause of death for 15 to 18 year-old Inupiat youth (5, 17). These Circumpolar regions have demographically young populations, which can lead to a higher burden on mental health (2, 7, 9). In Northern Canada, more than half of the Inuit population is under the age of 24 (11, 18), a reality also reflected in Circumpolar communities across Greenland, Norway, and Alaska (1, 3, 11, 16).

Mental health issues must, however, be considered within a larger socio-cultural history (7, 12, 19, 20). Arctic communities have experienced significant social and economic transitions and transformations over the last 50 years, stemming from rapid changes in lifestyles and livelihoods across the Arctic – events which today contextualize youth mental health (4, 6–9, 12, 13, 21). Circumpolar communities also experience inequalities in housing, healthcare, education and employment when compared to non-Indigenous populations (4, 6, 7, 9, 11, 22–27), which further impacts youth mental health. Many Arctic Indigenous youth are growing up in a context very different from their parents and grandparents, are facing new challenges, and are having to navigate multiple and often competing worlds and value systems (9). Environmental change is also having serious and often negative impacts in Arctic regions, with implications for lifestyle, livelihoods, culture, health and well-being widely documented (21, 24, 26, 28–36). Most research suggests a connection between deteriorating environmental conditions that disrupt livelihoods and ways of life, along with poor basic human infrastructure, as being major contributing factors to the poor mental health record (2, 23, 26, 27, 37).

Most mental health research on Indigenous youth has focused on the prevalence of mental health challenges and outcomes, such as suicide rates, and their individual risk factors, such as substance abuse, previous mental health disorders, and family conflict (2, 19, 38, 39). More than a decade ago, however, Kirmayer et al. (23) recognized the need to address the lack of research on factors promoting well-being and resilience among Indigenous youth. Since then, a small but growing body of work has begun to identify possible protective factors for Indigenous mental health. This research calls for further work to articulate the nuanced *mechanisms* and *pathways* through which protective factors may contribute to community and individual well-being (6, 7, 8, 9, 14, 19, 20, 40), and do so from the perspective of local perceptions of and approaches to mental health and well-being (41, *cf*. 12,42).

Given that mental health resilience will differ within and among communities, it is crucial to take into account the diverse cultural, geographic, political, economic, and social settings, contexts and histories when considering resilience in an Indigenous context, particularly when considering the diverse Circumpolar Indigenous populations (4, 6, 7, 12, 13, 21, 33, 43). For example, from an Inuit holistic perspective, health and well-being are just as much dependent on the physical, spiritual and social environment as they are on individual circumstances (12, 27, 33). Although past meanings of resilience for Arctic Indigenous populations related to their resourcefulness and adaptability to the unpredictable Arctic environment, in the present day, the meaning of resilience has shifted to also encompass the ability to adapt to challenging and rapidly changing physical, cultural, political and socio-economic environments, and includes an understanding of the importance of community cohesion, overall health, spiritual traditions and cultural connectivity (4, 7, 12, 21, 26, 27, 33).

Despite the importance of and the need for research on this topic, there has been, to our knowledge, no synthesizing review that identifies the protective factors specifically for Circumpolar Indigenous youth mental health or that examines causal mechanisms and pathways. This is an important research gap that needs to be addressed if we are to enhance and develop appropriate and effective health programming and resources to deal with the mental health disparities in Circumpolar countries (6, 7, 14, 19, 40, 43). In this context, this literature review asks the question

What are the protective factors that support and promote Circumpolar Indigenous youth mental health resilience and why do these factors enhance resilience?

## Methods

This systematic literature review uses the Preferred Reporting Items for Systematic Reviews and Meta-Analyses (PRISMA) guidelines (44) and draws on components of a realist review (45). Systematic reviews provide a replicable, rigorous and organized approach to evaluating and synthesizing research findings across studies, disciplines and approaches (46, 47). Realist reviews delve further into the questions of why these protective factors work, how they work, for whom and in what context (45). Realist reviews extend systematic reviews by moving beyond reporting outcomes to explaining the underlying causal mechanisms for these outcomes (45). The Circumpolar Indigenous youth populations considered in this review live in particular geographic contexts and, as such, a realist approach is especially appropriate as it places emphasis and importance on understanding context to explain how and why certain outcomes arise from specific places or within certain groups or environments (45). This style of literature review (i.e. systematic realist review) is being consistently used within social sciences in areas such as climate change adaptation, food security, health geography and international development (48–52) and has been successfully employed in Circumpolar mental health contexts before (2).

### Search strategy

**Fig. 1:**
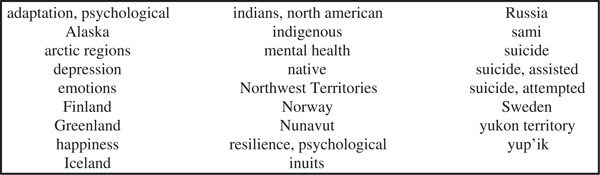
Medical Subject Headings (MeSH) used for database search in MedLine.

**Fig. 2:**
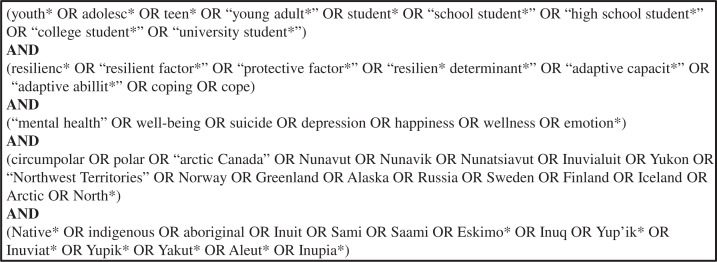
Search equation used in PubMed and Web of Knowledge based on MeSH, test searches, and consultation with research librarian.

Three electronic databases – PubMed, Web of Knowledge and Medline – were used to search for English-language, peer-reviewed published literature to identify the protective factors that may promote resilience in Circumpolar Indigenous youth populations to mental health problems. Several test searches were conducted in the selected databases to experiment with various synonyms and refine the subject headings and search string to get the most relevant and comprehensive search results. Consultation with an academic research librarian also informed the final search terms. Once finalized, the initial literature search was conducted through a 2-step process. First, Medical Subject Headings (MeSH) were used for the search in Medline to get an initial sense of the literature in the field (Fig. 1). Second, a search string informed by the initial MedLine search was applied to the PubMed and Web of Knowledge databases (Fig. 2).

All returned articles were uploaded to EndNote X5^®^, a reference management software programme, and duplicates were removed. A 2-stage screening process was then used to remove irrelevant articles, beginning by screening of titles and abstracts with reference to inclusion and exclusion criteria (Table I), followed by in-depth review where necessary. If articles did not meet one or more of the inclusion criteria, then they were not included for review (for example, if an article discussed Circumpolar Indigenous youth mental health with respect to risk factors and had no discussion on protective factors then it was not included).^1^ Many of the excluded references, however, were helpful in providing context and framing for this article. Before conducting the literature search, adding a criterion to the inclusion and exclusion criteria that would exclude empirical articles and provide more congruity between articles was considered. However, adding this criterion significantly limited the number of relevant articles and would have decreased the number of articles for review. To capture a reasonable and representative body of literature, both empirical and theoretical articles were included. Forward and backward citation tracking was then conducted to ensure all relevant articles were captured in the search. Finally, a snowballing technique was also applied, which involved emailing 3 key authors working in the field of Circumpolar youth resilience and mental health to inquire about their research and other relevant publications that may not have been found in the electronic search and to request clarification or full-text files of their publications if not available online. Snowballing and citation tracking confirmed that the database searches were thorough.

**Table I:** Table of inclusion and exclusion criteria used in determining which articles were applicable to review

Inclusion criteria	Exclusion criteria
English-language source	Non-English-language source
Peer-reviewed journal articles (empirical or theoretical)	Books, reviews, editorials, conference proceedings, commentaries
Anything published up until 25 September 2013	N/A
Focus on (or distinction of) an Indigenous population (i.e. Inuit, Yup'ik, Inupiat, Sami)	Population included in study is not Indigenous or there is no distinction between Indigenous and non-Indigenous populations
Include youth as only population of study or as a distinct group in study participants with specific discussion/coverage on youth	Youth are not included in study or are only briefly mentioned
Include at least 1 paragraph on resilience/protective factors that enhance mental health or make explicit reference to certain protective factors	Only focus on risk factors and no mention of resilience/protective factors
Circumpolar regions/countries only (Canada, Greenland, Norway, Russia, Finland, Iceland, Sweden or Alaska, USA)	Non-Circumpolar countries
N/A	Duplicate of previously found article

### Data extraction

A codebook was created to extract specific information from each article for synthesis. Information collected included general aspects of the study (e.g. first author, year, objective and study design) as well as specific questions being asked of the literature (e.g. What are the protective factors identified? How are causal mechanisms or explanations about the protective factors described? What policy recommendations are made by the authors?). A section on methodology, study design, measures used, and other method notes was also included in the codebook to track the type of article (i.e. empirical or theoretical) and document the source of the information^2^ (Table II).

**Table II:** Categories of information extracted from articles including general aspects and specific questions

Data extraction categories	Questions asked
Article information	First author
	Year
	Journal
Geographic	Study site – community, state/territory/province, country
	Objectives
	Methodology/study design
	Measures used
	Other methods notes
	Timeframe of study
Study population	Indigenous group (Yu'pik, Sami, Inuit)
	Size of study population
	Age
	Gender considered
Protective factors and causal mechanisms	What are the protective factors identified?
	Why? Pathways through which these factors protect/increase resiliency
	Mental health behaviours discussed (outcomes to avoid)
	Key results
Wording	Explicitly stated that these are protective factors? (wording)
	Is the word “resilience” used in the article?
Recommendations and take home messages	Recommendations
	Intervention/prevention thoughts?
	Strategies, interventions, or best practices within communities that promote strong mental health in the younger generation?
	Are the recommendations based on protective factors?
	Lessons that can be applied to future Indigenous youth-related mental health research and work?
Other	Other notes
	Introductory material useful to setting the stage
	Weaknesses – what was not included?

Given the small sample size, the use of statistics to examine publication trends, resilience factors and causal mechanisms was precluded. Instead, the analysis focuses in depth on the factors enhancing the mental well-being of Indigenous youth. This is consistent with other realist and systematic review papers also based on a small sample of publications (2, 49).

## Results

### Limited research has been conducted but is expanding

**Fig. 3:**
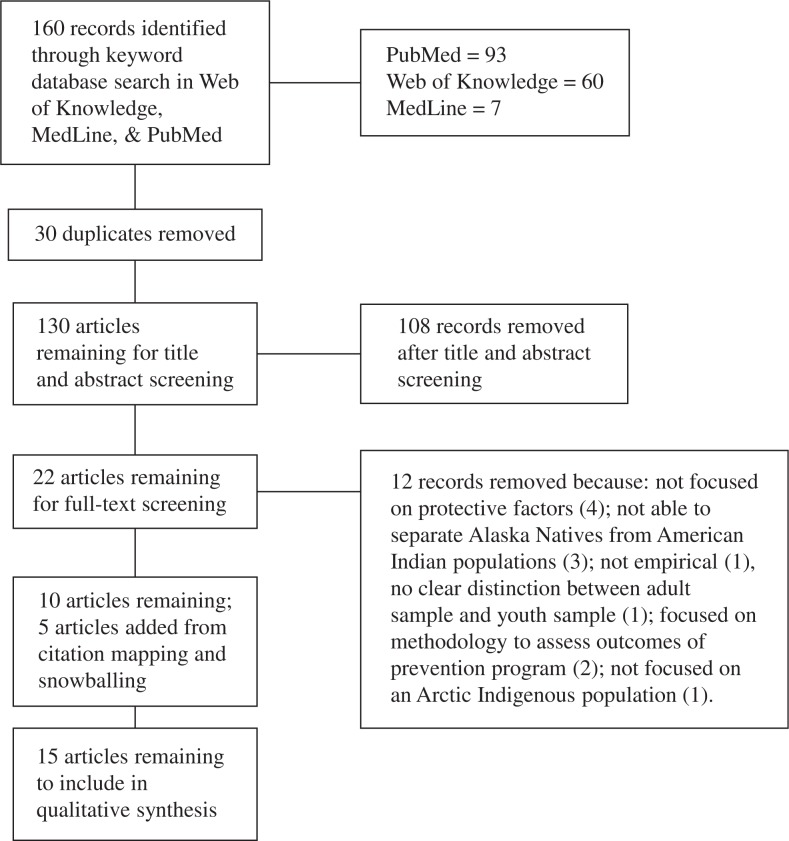
Search strategy and results.

Of the 160 records collected from the database searches, 30 duplicates were removed leaving 130 articles for initial screening of title and abstract. From this screening, 108 articles were excluded (Table III). Twenty-two had a full-text screening and 12 were excluded. Of these 12, 4 did not focus on protective factors, 3 did not separate Alaskan Natives from American Indian populations, one was not empirical, one did not make a clear distinction between the adult sample and youth sample, 2 focused on methodologies utilized to assess outcomes of prevention programmes and one was not focused on an Arctic Indigenous population. In addition to the 10 remaining articles, 5 were added after citation mapping, which left a total of 15 articles to be reviewed (Table IV, Fig. 3). All reviewed articles cover only 4 of the Circumpolar countries (Canada, Norway, Greenland and Alaska, USA) and only 4 Indigenous groups [Inuit (Greenlandic and Canadian), Sami, and Alaskan Natives] (Table IV).

**Table III:** Breakdown of articles excluded in title and abstract screening

Inclusion criteria not met	Number of articles
Not youth	6
Not Indigenous	3
Not Arctic	35
Not youth or Indigenous	1
Not Arctic or Indigenous	6
Not youth or Arctic	9
Not youth, Indigenous, or Arctic	5
Not mental health	4
Not protective factors	4
Not Indigenous or mental health	1
Not at all related	14
Not Arctic, youth, mental health	8
Not Indigenous, youth, mental health	3
Not Arctic or mental health	7
Not youth or mental health	1
Not Arctic, Indigenous, or mental health	1

**Table IV:** List of final 15 articles included in literature review with the Indigenous group studied and the location of research

Articles reviewed	Indigenous group	Location
Allen, J., G. V. Mohatt, et al. (2006). “The Tools to Understand.” *Journal of Prevention & Intervention in the Community* 32(1–2): 41–59.	Alaska Natives	Alaska (USA)
Bals, M., A. L. Turi, et al. (2010). “Internalization symptoms, perceived discrimination, and ethnic identity in indigenous Sami and non-Sami youth in Arctic Norway.” *Ethnicity & Health* 15(2): 165–179.	Sami	Northern Norway (Finnmark, Troms, Nordland)
Bals, M., A. L. Turi, et al. (2011). “The relationship between internalizing and externalizing symptoms and cultural resilience factors in Indigenous Sami youth from Arctic Norway.” *Int J Circumpolar Health* 70(1): 37–45.		
Bals, M., A. L. Turi, et al. (2011). “Self-reported internalization symptoms and family factors in indigenous Sami and non-Sami adolescents in North Norway.” *Journal of Adolescence* 34(4): 759–766.		
Decou, C.R., M.C. Skewes, et al. (2013). “Traditional living and cultural ways as protective factors against suicide: perceptions of Alaska Native university students.” *Int J Circumpolar Health* 72:20968.	Alaska Natives	Alaska (USA)
Ford, T., S. Rasmus, et al. (2012). “Being useful: achieving indigenous youth involvement in a community-based participatory research project in Alaska.” *Int J Circumpolar Health* 71(0): 1–7.	Alaska Natives	Southwest Alaska (USA)
Kirmayer, L. J., M. Malus, et al. (1996). “Suicide attempts among Inuit youth: A community survey of prevalence and risk factors.” *Acta Psychiatrica Scandinavica* 94(1): 8–17.	Inuit	Nunavik, Northern Quebec (Canada)
Kirmayer, L. J., L. J. Boothroyd, et al. (1998). “Attempted suicide among Inuit youth: Psychosocial correlates and implications for prevention.” *Canadian Journal of Psychiatry-Revue Canadienne De Psychiatrie* 43(8): 816–822.		
Kral, M. J., L. Idlout, et al. (2011). “Unikkaartuit: meanings of well-being, unhappiness, health, and community change among Inuit in Nunavut, Canada.” *Am J Community Psychol* 48(3–4): 426–438.	Inuit	Igloolik and Qikiqtarjuaq, Nunavut (Canada)
Mohatt, G., S. M. Rasmus, et al. (2004). “‘Tied together like a woven hat:’ Protective pathways to Alaska native sobriety.” Harm Reduction Journal 1(1): 10.	Alaska Natives	Alaska (USA)
Spein, A.R., C.P. Pedersen, et al. (2013). “Self-rated health among Greenlandic Inuit and Norwegian Sami adolescents: associated risk and protective correlates.” Int J Circumpolar Health 72:19793.	Inuit and Sami	Greenland and North-Norway
Wexler, L. and B. Goodwin (2006). “Youth and adult community member beliefs about Inupiat youth suicide and its prevention.” Int J Circumpolar Health 65(5): 448–458.	Inupiat	Alaska (USA)
Wexler, L., K. Jernigan, et al. (2013). “Lived Challenges and Getting Through Them: Alaska Native Youth Narratives as a Way to Understand Resilience.” Health Promotion Practice DOI: 10.1177/1524839913475801.		
Wexler, L. (2013). “Looking across 3 generations of Alaska Natives to explore how culture fosters indigenous resilience.” Transcultural Psychiatry DOI: 10.1177/1363461513497417.		
Wexler, L., L. Joule, et al. (2013). “‘Being responsible, respectful, trying to keep the tradition alive:’ Cultural resilience and growing up in an Alaska Native community.” Transcultural Psychiatry DOI: 10.1177/1363461513495085.		

Of the 15 included articles, several first authors are also co-authors for one or more of the other articles reviewed (e.g. L. Kirmayer, J. Allan, G. Mohatt, and L. Wexler), while most papers cite at least one of the other 8 first authors, with several instances where studies use the same data set. For instance, all 3 of Bals’ publications (43, 53, 54) use the same data set, while Mohatt et al. (41) and Allen et al. (16) are reporting different aspects of the same project. The more recent research is intentionally building on what has already been done [e.g., Decou et al.'s (14) study utilizes the findings of Allen et al. (16) to inform their research focus and interview questions]. This illustrates the small number of researchers approaching Indigenous youth mental health from a protective factor and resilience lens.

Research in this field is also recent. More than half of the articles were published in the last 3 years with 5 published in 2013 alone. Kirmayer et al. (10) was the first to include protective factors in publications about Indigenous youth mental health research in the Circumpolar North in the late 1990's, although this work still primarily focused on risk factors for suicide among Inuit youth in Canada. Mohatt et al.'s (41) article focusing specifically on protective factors for youth resilience was published almost a decade later. Going further, the most recent work in this field recognizes and is pursuing the crucial identification and understanding of causal pathways and mechanisms and has begun to investigate locally appropriate, youth-driven resilience strategies and initiatives in Circumpolar Indigenous communities (7–9, 14). As the articles in this review demonstrate, the transition to emphasizing resilience and supporting protective factors has been called for and is now occurring in emerging literature (7–9, 14).

### Protective factors are key to youth resilience

There are more than 40 protective factors which are catalogued as community level (Table V), family level (Table VI) and individual level (Table VII). Most protective factors are reported by multiple studies, including community factors such as positive role models within the community, and traditional knowledge and practice; family factors such as kinship; and individual factors such as belief in self and desire to be useful and to contribute (3, 7–9, 11, 14, 16, 17, 41, 43, 53, 54). The categorization in the 3 levels and some of the reported protective factors are similar to results from resilience research with youth (both Indigenous and non-Indigenous) in other regions, such as the individual factors of belief in self, academic achievement and feeling useful; family factors in terms of the quality of the parent–child relationship; and community factors including supportive environment, extracurricular opportunities and participation, and role models (3, 7, 9, 10, 13, 14, 38, 39, 41). Factors that are specific to the Indigenous Circumpolar context include continuous communication and interaction, cultural revitalization, ethnic socialization at home, kinship structure, traditional knowledge, ethnic pride, and mindfulness and awareness of the consequences of one's actions.

**Table V:** Community-level protective factors identified in literature review

Protective factors	Authors who identified the protective factor	
Positive role models	Allen et al., 2006	Mohatt et al., 2004
	Wexler and Goodwin, 2006	Wexler, Joule, et al., 2013
Sense of collective responsibility and	Allen et al., 2006	Ford et al., 2012
community connectedness	Mohatt et al., 2004	Wexler, 2013
	Wexler, Jernigan, et al., 2013	
Sense of belonging in community	Wexler, 2013	
Meaningful opportunities to be involved within	Allen et al., 2006	Ford et al., 2012
community or school community	Mohatt et al., 2004	Decou et al., 2013
Community-wide limits/standards/expectations	Allen et al., 2006	Mohatt et al., 2004
Safe places	Allen et al., 2006	Mohatt et al., 2004
Supportive, caring, encouraging, cohesive communities	Mohatt et al., 2004	Wexler and Goodwin, 2006
that show concern and reach out to youth	Decou et al., 2013	Wexler, Jernigan, et al., 2013
Strong relationships with community members	Kirmayer et al., 1998	Wexler and Goodwin, 2006
(peers or other adults)	Decou et al., 2013	Wexler, Jernigan, et al., 2013
	Wexler, Joule, et al., 2013	
Mentorship from older generations	Wexler, Jernigan, et al., 2013	Wexler, Joule, et al., 2013
Continuous communication, talking, and interaction	Kral et al., 2011	Wexler and Goodwin, 2006
Regular church attendance	Kirmayer et al., 1996	Kirmayer et al., 1998
Community control	Kral et al., 2011	
Cultural revitalization	Bals et al., 2010	
Community recognition, respect, and appreciation	Wexler, Jernigan, et al., 2013	

**Table VI:** Family-level protective factors identified in literature review

Protective factors	Authors who identified the protective factor
Close relationship with parents	Allen et al., 2006
	Mohatt et al., 2004
	Spein et al., 2013
Affection and praise	Allen et al., 2006
	Mohatt et al., 2004
	Wexler, Jernigan, et al., 2013
Models of sobriety and safe/protective family environment	Allen et al., 2006
	Mohatt et al., 2004
Transmission of expectations and values	Allen et al., 2006
	Mohatt et al., 2004
Family history of having received treatment for psychiatric problem	Kirmayer et al., 1996
Parental approval of friends	Bals et al., 2011b
Sense of being treated as special/being valued	Allen et al., 2006
	Mohatt et al., 2004
Kinship structure (i.e. family connectedness and importance of	Bals et al., 2010
extended family and adopted kin)	Bals et al., 2011b
	Kral et al., 2011
	Mohatt et al., 2004
	Wexler, Jernigan, et al., 2013
	Wexler, Joule, et al., 2013
Native language learned at home and competence in native language	Bals et al., 2010
	Bals et al., 2011b
Ethnic socialization at home	Bals et al., 2010
	Bals et al., 2011b

**Table VII:** Individual-level protective factors identified in literature review

Protective factors	Authors who identified the protective factor	
Belief in self	Allen et al., 2006	Bals et al., 2010
	Mohatt et al., 2004	
Sense of purpose	Wexler, 2013	
Physically being in home community	Wexler, 2013	
Wanting to contribute, be useful to others, take care of others,	Allen et al., 2006	Ford et al., 2012
and give back to the community (i.e. to be a role model)	Mohatt et al., 2004	Wexler, Joule, et al., 2013
	Wexler, Jernigan, et al., 2013	
Mindfulness and awareness of the consequences of one's individual actions upon the community	Allen et al., 2006	Mohatt et al., 2004
Reflection	Mohatt et al., 2004	
Sense of responsibility to oneself, family, and/or community	Allen et al., 2006	Mohatt et al., 2004
	Wexler, Jernigan, et al., 2013	Wexler, Joule, et al., 2013
Learning values of harmony and co-operationas well as autonomy and hardiness	Bals et al., 2011a	
High level of academic achievement	Kirmayer et al., 1996	Spein et al., 2013
Ethnic pride	Bals et al., 2011a	Ford et al., 2012
	Wexler, Jernigan, et al., 2013	
Cultural/ethnic identity and/or affiliation	Wexler, 2013	Wexler, Joule, et al., 2013
Traditional knowledge, cultural values, and practice	Bals et al., 2010	Bals et al., 2011a
(e.g. eating country foods, being out on the land,	Kirmayer et al., 1998	Kral et al., 2011
doing subsistence activities, attending tribal events,	Decou et al., 2013	Wexler, Jernigan, et al., 2013
listening to traditional stories)	Wexler, Joule, et al., 2013	Wexler, 2013
Systems of reciprocity and reciprocal bonds	Wexler, Jernigan, et al., 2013	Wexler, Joule, et al., 2013
Physical activity and active lifestyle	Spein et al., 2013	Decou et al., 2013
Staying busy	Wexler, Jernigan, et al., 2013	
Self-reliance (e.g. seeking support from a friend, keeping a journal, creatively handling problems)	Wexler, Jernigan, et al., 2013	Wexler, Joule, et al., 2013
Being committed to community and culture	Wexler, 2013	

Adults and youth differ in their perspectives on youth suicide prevention (17). Adults feel that the most effective prevention is to offer activities and education, and create a sense of culture; youth highlight the need for adults to simply talk to them about their everyday lives and their futures, and offer guidance, support and companionship (17). The benefit of voluntary, informal interactions between youth and adults was also echoed by other authors (11).

Many protective factors overlap the 3 categories of individual, family and community levels and many are directly connected to other factors (7, 9, 11, 14, 41, 54). For example, the Inuit practice of hunting is a community activity, but directly links to sharing and eating country food, which are family and individual factors, respectively (11). Other individual protective factors that are fostered through hunting and spending time out on the land include belief in self, self-reliance, mindfulness and awareness of the consequences of one's actions, self-confidence and sense of purpose (7, 9, 11, 41, 54). Participating in subsistence activities also provides opportunities to be involved in the community, receive mentorship from older generations and build relationships that contribute to community connectedness – all examples of the ways in which community factors can foster other community factors (3, 7, 9, 14, 16, 41). Another example is kinship, which is intertwined with cultural identity and may create a sense of security and strong cohesive families and communities; this, in turn, may provide a strong social support network and individual sense of belonging (7, 8, 9, 41, 53, 54). In this sense, protective factors work together in complex relationships to foster or enhance other protective factors to create an environment that supports healthy youth development, promotes feelings of competence and fosters a positive attitude towards problem solving and pursuing meaningful and realistic strategies to enhance well-being (7–9).

### Identifying causal pathways and protective mechanisms is essential

Several authors identified the lack of nuanced insight on the causal pathways or mechanisms through which protective factors enhance mental health (7–9, 14, 16, 41, 43). These authors point to 3 main reasons that protective factors exert a positive influence on mental health: they contribute to developing a supportive social environment; they enhance self-esteem and self-confidence and foster self-reliance; and they enable individuals to participate in their land-based culture (Table VIII) (7–9, 14, 16, 41, 43).

**Table VIII:** Causal pathways identified for protective factors around one's social environment including family, peer, and community relationships

Protective factor	Causal pathway	First author
Kinship	Extended family can provide support in the event that the immediate family cannot, sense of connection to others beyond the immediate family.	Bals (2010, 2011b)
	Spending time with family and kin allows for opportunities to learn culture.	Kral (2011)
Close relationship with peers	Allows opportunity for youth to take on adult-like roles and offer support, be dependable, responsible, and responsive to others. Provides a chance to develop awareness of others and to also receive support from peers.	Wexler, Jernigan (2013) Wexler, Joule (2013)
Social Network (Includes relationships with family, peers, and community members)	Relationships and friendships can mediate access to cultural and material assets and thus increase one's capacity (e.g. kin, adopted kin, or friends can provide support resources like a skidoo available to get out on land). These relationships also provide a platform for youth to build/construct their identity and resilience.	Wexler, Jernigan (2013) Wexler, Joule (2013)
Mentorship from older generations	Source of support and guidance in how to handle problems. Provides examples to youth of how to get through difficulties while instilling belief in youth that they can also get through difficulties like their mentors and ancestors.	Wexler, Jernigan (2013)

First, the importance of youth having opportunities to participate in a range of social activities within supportive environments is recognized as key to mental health resilience. Kirmayer et al. (10) identify that regular church attendance acts as a protective factor, not because of adherence to the beliefs or practices of the religion, but rather because attending weekly services is an opportunity to strengthen social ties and engage with the members of the church community. He generalizes this explanation to include other community activities or involvement (10). Other authors also emphasize the importance of community connectedness and a sense of belonging (3, 7, 8, 16, 41). Broadly, family and community protective factors create, and subsequently impact, a larger cohesive social environment that supports youth, protects against trauma and promotes reflection (8, 41). The strong relationships, supports, and role models that create this social environment also interact with individual protective factors and are influential during times of personal reflection and when making important life decisions (9, 41). For example, youth who have more relationships with friends, extended family, and other community members have more access to resources providing more opportunities to navigate challenges (e.g. accessing a skidoo to get out on the land) and thus increases adaptability (7, 9). The relationships and resources available to youth structure and shape not only their approaches to problem solving but also mediate other protective factors such as mentorship and opportunities to be involved (7–9, 14) (Table IX).

**Table IX:** Causal pathways identified for various protective factors

Protective factor	Causal pathway	First author
Regular church attendance	Participation in a community activity, engage in community and family networks, strengthens social ties, and builds strong social support. Also may offer consolation and hope in difficult times.	Kirmayer (1998)
High academic achievement	Source of self-esteem, builds problem-solving skills, presents hope for future.	Kirmayer (1996)
Awareness, mindfulness, and reciprocity of action	A mechanism in and of itself – individuals who are socialized in this context are more sensitive to the effects of their behaviours on the whole, and draw strength from the whole.	Mohatt (2004)
Sense of being treated as special/important	Encourages youth to live up to high standards and be responsible for themselves and others.	Mohatt (2004)
Opportunities (in the community, at school, with research project)	Opportunities to be involved with research as co-researchers engages and empowers youth, giving them sense of control and ownership. Praise from adults and elders gives youth pride. Research process offers space to talk to peers, reflect on mental health problems, and learn about oneself. Youth-researcher partnerships also increase egalitarian relations between young people and adults.	Ford (2012)
	Some activities, such as sport teams, provide incentive to do well in school (e.g. must do well in classes to participate in sport team).	Wexler, Jernigan (2013)
Self-reflection	Often leads to a conscious decision (i.e. to not drink or to drink responsibly)	Allen (2006)
Being responsible	Through activities like fixing something, doing homework, watching siblings, raising money for community, or doing chores, youth have a chance to contribute in a meaningful way and gain a sense of purpose and personal well-being. Also demonstrates autonomy and community connectedness.	Wexler, Jernigan (2013)
Being useful	Leads to feeling responsible and creates systems of reciprocity and availability to help one another which leads to young people having someone to talk to in difficult times such as times of loss. Reflects Indigenous values.	Wexler, Jernigan (2013)
Physical activity	Positively influences self image, family, and peer relationships, and general well-being among youth.	Spein (2013)
Sense of belonging in home community	Through a sense of belonging in one's home community youth feel more connected to their culture.	Wexler (2013)

Note that not all protective factors were linked to a causal pathway.

Second, belief in oneself is identified as a protective factor and as a mechanism through which other protective factors function (16, 41, 43). For example, high academic achievement and learning, and practicing culturally significant activities are both documented to lead to greater self-confidence and self-esteem. Self-esteem, in turn, enhances mental health and well-being (10, 11, 43). Similarly, traditional knowledge and the ability to practice cultural activities have also been identified as mechanisms that promote a sense of self and self-esteem (7, 9, 11, 14, 16, 41, 43, 53). Bals et al. note that speaking the Sami language leads to feeling accepted by other Sami people and contributes to a sense of self as part of the Sami population (53). The ability to hunt and knowledge of the land were also described as factors that enhance Indigenous youth self-esteem and well-being (11, 16) (Table X).

**Table X:** Causal pathways identified for protective factors around culture including practicing traditional activities and having a positive ethnic identity

Protective factor	Causal pathway	First Author
Learning and practicing culture (e.g. traditional practices and subsistence activities)	Strengthens self-esteem, ethnic identity, and self-regulation skills. Sharing cultural knowledge enhances in-group cohesiveness and support through experience of shared meaning-making. Also provides an opportunity to participate in the passing down of traditional knowledge and cultural values thus creating feeling of keeping culture alive. Enhances relationships between youth and the land and between youth and their families. Facilitates and creates context for important mentoring relationships and connection to older generations leading to youth feeling supported. Promotes healthy living, facilitates involvement and creates opportunities for sharing. Gain new skills (e.g. hunting, camping, riding) and knowledge. Develops sense of ethnic pride. Provides a meaningful way for youth to contribute and give back to the community, earn respect for skills, and feel appreciation from community (e.g. through sharing kill). Subsistence skills demonstrate strength and survival and are associated with ability to respond and to be resilient in face of hardship. Being out on the land requires youth to act selflessly, be responsible and less petty, show respect, rely on others, and distinguish between what is essential vs. trivial. Meaningful engagement in traditional activities also contributes to sense of purpose, feeling more self efficacious, and staying busy. Traditional activities associated with fulfilment, sense of calm, and sense of being special. Shows cultural continuity with one's heritage and connects youth to sense of how life used to be. Provides time with parents to learn skills and how to be in the world. Ability to practice culture is source of pride and well-being.	Bals (2010, 2011a) Decou (2013) Wexler, Jernigan (2013) Wexler, Joule (2013) Kral (2011)
Positive cultural/ethnic identity and shared heritage	Leads to and increases self-esteem, feelings of self-worth, self-efficacy, connectedness, commitment, and purpose. Provides sense of belonging. Offers perspectives to draw from to overcome challenges and be well. Evokes sense of strength and capability. Creates larger shared context in which youth can situate themselves and their struggles in relation to others, to their history, and to a collective. Provides a means to structure one's understanding and ideas of their role in the world (e.g. as a youth, as Inuit/Sami/Inupiat)	Wexler (2013) Wexler, Joule (2013) Allen (2006)
Ethnic socialization	Influence on interpersonal and intra-psychic processes, increases self-confidence, develops positive attitudes towards ethnic identity, teaches self-regulation and coping skills.	Bals (2010, 2011b)
Native language	Personal and relational significance – important part of ethnic identity that strengthens in-group cohesion, personal pride, and sense of history and culture.	Bals (2010, 2011b)

Third, the importance of learning and participating in one's culture is acknowledged by all authors as a protective factor but is perhaps best exemplified through the Sami in Norway. Similar to all Circumpolar Indigenouspopulations, the Sami have a history of colonization, which began in 1830, and included assimilation through residential schools, language prohibition and loss of cultural practices (13, 21, 43, 53, 54). The suicide rates for Sami youth were higher than the non-Sami average in the 1980's. Since then, however, the situation has improved substantially, and recent research indicates that currently, there are little to no disparities in health and well-being between Sami and non-Sami in Norway (53). This can, in part, be explained by the past 30 years of explicit and conscious political, cultural and language revitalization efforts in Norway, which includes the development of many Sami institutions (i.e. schools, hospitals, a Parliament, and a University), a high degree of self-governance, good living conditions, increased socio-economic status and positive socio-cultural development (1, 13, 21, 53, 55). These efforts and developments have contributed to supporting the Sami lifestyle and culture allowing Sami youth more opportunities to learn and participate in their culture, as well as to assisting in improving health outcomes for this population (13).

Considering the limited literature exploring causal pathways and mechanisms through which protective factors enhance resilience, many authors called for more research and exploration into how protective factors enhance resilience (7–9, 14). These authors point to the importance of this knowledge and understanding for developing relevant and effective intervention and prevention programming (7–9, 14). Some authors were quite specific about where future research needs to be directed: Decou et al. (14), for example, call for further research to explore the pathways through which traditional activities and subsistence living can contribute to suicide prevention.

### Community and culture are at the heart of youth mental health

The importance of community and culture in enhancing and expanding mental health are 2 key themes of all the articles in this review. All authors place emphasis on establishing and maintaining healthy relationships with family and community members, while simultaneously nurturing culturally specific relationships with the land, animals and plants, and developing cultural-based skills and activities (3, 7–11, 14, 16, 17, 41, 43, 56). More specifically, a sense of belonging to caring and supportive communities and families is crucial to making good decisions and to leading a healthy life (41, 43, 54). Physically being in one's home community emerged as a protective factor from one of the most recent articles in which Indigenous youth who were studying outside of their home community participated (8). These youth felt that they had to return home to feel better or heal from bad experiences while living away from their community (8). Although Elders interviewed in the same study described their experiences away from home as similar to the experiences described by the youth, the difference between the 2 generations was that Elders used their cultural identity to situate themselves in a larger shared context and collective, which led to feelings of support and belongingness when away from home (8).

All authors note the important role that culture plays in the lives of youth in Circumpolar Indigenous communities; however, some simply identify “Indigenous culture” as a protective factor in and of itself (54), while others note specific aspects of the culture that have protective features (7, 9, 11, 14). In Norway, Bals et al. (54) suggest that the Sami culture provides socialization, pride, and support and creates a resilience to protect Sami youth from depression and anxiety. In Canada, Kral et al. (11) give several examples of culturally specific protective factors: kinship structure, the tradition of visiting, going out on the land with family and friends, hunting and trapping, and sharing and eating country food. These individual protective factors are part of Inuit culture and associated with wellness, happiness, health and healing (11), as well as contributing to a sense of belonging, a basic human need, and a cross-cultural resilience factor for coping in times of stress (57, 58). Furthermore, in the articles by Mohatt et al. and Allen et al., the authors indicate that some of the more general protective factors, such as affection and praise, are important because they function within culturally specific contexts (i.e. certain ways of showing praise and affection can be culturally specific and lend a sense of connection and cohesion) (16, 41).

A few of the studies reviewed indicated that the youth felt their culture is being lost (8, 14). Several reasons behind these feelings expressed by participants included loss of native language, changing relationships with the land, changing relationships between families, diminishing traditional activities in daily life and decreased knowledge transmission between generations, a decline of traditional values, and less cultural participation (8, 14). Youth reported that the feeling of losing culture may be attributed to apathy or time constraints, but authors speculated that it may go deeper and be connected to the way youth are defining and understanding culture (i.e. as a defined set of activities and knowledge rather than a larger shared context and collective) (8), or may stem from comparisons with today's way of life to that of Elders and ancestors who lived off the land (8, 14).

## Discussion: Circumpolar youth mental health and well-being

This review intentionally focuses on literature examining mental health protective factors and resilience, rather than on articles that solely discuss risk factors. Although research on the prevalence of mental health problems and risk factors is important, it is equally valuable to identify what makes individuals resilient to these mental health problems to inform prevention strategies and foster healthy youth populations (7, 14, 20, 33, 40). A focus on resilience also captures physical, mental, emotional and spiritual characteristics of both individuals and communities, which is more reflective of Indigenous communities’ approaches to and understandings of health in the Circumpolar North (1, 12, 21, 27, 33). Despite the serious mental health issues facing Indigenous youth throughout the Circumpolar North, there is only a small and relatively recent body of research focused on understanding how to foster resilience to address these mental health disparities (7).

Mental health is not only an outcome in and of itself, it is also a determinant for overall community health and cohesion, and is directly and intimately tied to other aspects of community health and well-being (59). For example, in some Arctic communities, researchers have pointed to the profound effect of high suicide rates on community life (5, 10–13). Many, if not most, people in these communities have lost one or more family members or friends to suicide and, as such, it is difficult to remain positive and supportive of others during times of grief and mourning (5, 10–12). The high rate of suicide also creates an environment within these communities where few are untouched by suicide, and in some cases, where suicide becomes almost normalized (*cf*. 5).

It is noteworthy to mention here that some Sami populations are an exception to the high suicide rate that is characteristic of many Circumpolar Indigenous populations (1, 2). The low levels of suicide in many Sami communities have been attributed to good living conditions and socio-economic status, positive socio-cultural development, a high degree of self-governance and support for the Sami culture, the preservation of traditional language, and the overall cultural revitalization that has occurred in Norway over the past 30 years (1, 13, 21, 43, 53). The mechanisms through which, and extent to which, cultural revitalization has been protective to Sami youth requires further research, and more importantly, an understanding of why the mental health disparities in the youth population have almost disappeared in Norway (53). Work with other Indigenous and cultural groups may provide some insights to answer these questions. Wexler (19) and Wexler et al. (20) draw from experience and research with Northern Canadian Aboriginal youth and Bosnian and Palestinian youth who experienced war, and propose that cultural identity and cultural affiliation create protective factors such as self-efficacy and connectedness while at the same time create collective meaning and offer a larger context within which youth can situate their personal experiences thus enhancing resilience (60–62).

Individual experiences of mental health problems can also create undercurrents of grief, anger, frustration and worry that can decrease the resilience of a population to cope with further mental health challenges (5, 11, 12). If trouble with youth reflect problems within their families and communities (5, 23, 63), then the individual youth-level mental health problems may be indicative or reflective of community-level problems. Conversely, community-level problems can also be indicative of individual mental health issues and can highlight areas of mental health challenges. The overlap and connectivity across and between individual, family and community levels align with the holistic perspective of well-being held by many Circumpolar Indigenous populations, in which important aspects of life and health are viewed as intertwined (1, 11, 12, 21, 27). This overlap and connectivity also reinforces the important role of community in the design and implementation of preventative strategies and interventions to enhance youth mental health (11, 12, 16, 17, 41, 56, 63).

The research reviewed here consistently identifies the importance of culture and community in enhancing youth mental health. Other related research also reports this relationship (*cf*. 19,20,40). For example, even when Indigenous youth are living away from their home communities, cultural identity and pride, a desire to contribute to their community, and community connectedness and relationships remain important to youth success in a different context (40). Although the ways in which culture was defined varied between authors and within study groups, and despite the difficulty that youth often experienced when trying to explain how culture enhances resilience, youth from all the studies were certain about the importance of culture for their overall well-being. In addition to the ambiguity of these terms, within these 2 themes, there are also gaps in understanding. For example, while this review illustrates that a supportive, caring and connected community can act as a protective factor and have a positive effect on individuals (11, 16, 17, 41, 56), it is less clear how an individual's mental or emotional situation can impact the community or how communities can build strength in difficult conditions. The only mention of the impact of youth on communities comes from Decou et al. (14) who state that positive and healthy youth will strengthen communities but no further elaboration is provided. Considering the challenging social and economic context of many Northern communities (1, 4, 5, 12, 13, 21), attention to the interaction between individual mental health and overall community wellness is also necessary.

Exploring the role communities can play in shaping youth resilience and adaptive capacities is a new trend that has emerged in the past year in published resilience research (7–9, 14). Among this emerging work is research with youth living away from home (9, 14). Despite wanting to stay in one's home community, leaving is viewed as essential to accessing more opportunities and feeling successful and capable whereas staying at home can lead youth to feel stagnant or limited because certain opportunities are only available in larger urban centres and not in one's home community (8, 9). Researchers are beginning to address this by partnering with communities to create opportunities for youth to be co-researchers and increase opportunities for youth to gain experience and learn new skills in their home community (3, 6, 8, 9, 11, 16, 41).

Suicide was also highlighted by many of the authors as an important consideration when examining youth resilience, yet differing perspectives on youth suicide prevention emerged (17). From the adult perspective, formalized, cultural-based programmes and activities are most effective for youth suicide prevention, whereas youth perceive informal interaction, guidance, support and companionship from adults and Elders as the best way to prevent youth suicide (17). These findings provide valuable information on which to build prevention programmes that incorporate youth ideas of appropriate resources and support, such as fewer structured programming pieces and more informal interaction between youth and adults. Some of Wexler's more recent work with Inupiat in Alaska indicates that older people struggle with knowing how to promote and support healthy and positive youth development in a modern context (5, 9). Future research on youth mental health and resilience, then, should not rely solely on adult perspectives of youth needs to inform mental health services; rather, research should intentionally and meaningfully include youth perspectives, narratives, values, ways of knowing, ideas and experience to provide accurate and holistic understandings of individual and community mental health issues and appropriate and relevant intervention and prevention programmes (6, 7). Some of the more recent research in this area highlights youth narratives (9), youth identified protective factors (14), and self-rated health and perceptions of well-being reported by young people (13) and, as such, provides useful examples of integrating youth perspectives into resilience research and work.

A concentration on the opportunities for youth to develop and foster healthy and positive relationships with adults to enhance their mental health resilience and adaptive capacities was another trend in the literature (11, 16, 17, 41, 56). While having healthy adult role models to provide care, guidance and cultural insight and skills is no doubt of great importance, it is interesting that these articles do not discuss or examine peer-to-peer support or the effects of having strong peer support on youth mental health. Further research examining the impacts of peer support networks on youth mental health, and analyzing the effects of mental health challenges within peer groups on overall group mental health and well-being, would be of interest to the Circumpolar regions.

Many of the articles illustrate the importance of youth feeling connected to their culture through relationships with Elders and seniors, which also points to the protective nature of informal communication, mentorship and of having positive role models (7, 9, 11, 17). Kral et al. (11) strongly emphasize that relational life is at the core of Indigenous identity (*cf*. 64) and, as such, meaningful, daily interactions and communication between youth and adults is critical to well-being. The need for communication is only discussed in the literature as a face-to-face activity (11, 17), overlooking the emergence of new technology and social media and the role it can play in building social networks, sense of identity or community cohesion. One example of the use of technology is from Allen et al. (16) who created a successful multimedia programme on sobriety with Alaska Native youth, which includes audio and visual narratives on CD-ROM with an accompanying booklet containing life history sketches of individual sobriety stories from research participants. However, with the rapid adoption of social media within many Circumpolar communities, the impact of this type of communication on mental health and well-being is important to explore. For example, could Facebook, Twitter or Google+ act as social support networks that could enhance youth mental health and resilience? Social networks such as Facebook have gained tremendous use in the North in recent years as important communication tools for families and friends, as well as a way to connect people across Northern communities (63, 65–67). The use of these social networks is certainly very complex, with multiple potential positive and negative effects (68–72). The possibility of these tools to connect to others, share support and enhance one's mental health and well-being, however, is important to investigate from social network and mental health perspectives. An understanding of the ways young people communicate as well as youth perspectives of mental health programmes is also crucial to the development of appropriate and beneficial mental health support programmes (11, 17).

The most recent work on Arctic Indigenous youth resilience has begun to tie research to practice and provide tangible and concrete recommendations for communities and health services working on promoting healthy youth development (7). Many of the articles from 2013 point to the importance of culturally relevant opportunities that create and foster protective factors in youth such as participating in traditional activities, being responsible, developing self-reliance and feeling useful to the community (7, 9, 14). Research findings of protective factors such as the desire of youth to be useful and contribute to their communities as well as the importance of cultural practices and traditional activities should inform the priorities of resilience programming and intervention or prevention efforts (7, 9, 14). Research with non-Arctic Indigenous populations has also reported relationships between cultural identity and traditional practices for enhancing mental health (60). In some Arctic communities, the opportunities to participate in traditional activities, share knowledge and learn new skills have decreased in the last 50 years (9) and, as such, Wexler (8) states that increasing opportunities for subsistence activities could be a point of focus for intervention strategies. This overlaps with other scholarship calling for such programmes, albeit in different contexts (e.g. climate change adaptation) (73, 74).

Several authors identify the importance of incorporating cultural histories and stories of resilience from the various Indigenous cultures that may serve to connect youth to a shared context from which they can draw strength, resources and skills, and in which they can situate themselves and their struggles in relation to others, to their history, and to a collective sense of culture and identity (8, 9). A strong understanding of the past can help to link youth to both the suffering and the strength of their ancestors, thereby providing a collective experience with which youth can relate and identify (8). Being culturally grounded can also promote flexible and versatile resilience strategies; resilience strategies, therefore, should support youth in connecting to their cultural past and present and encourage youth to feel part of a larger collective with shared values and traditions to draw from in times of struggle and difficulties (8, 9).

Despite the challenges and risk factors facing Arctic Indigenous youth, authors who have focused on resilience indicate that the majority of youth in their study communities are resilient (7, 9, 14). Indeed, Arctic Indigenous populations have legacies of adaptability, creative responsiveness, and resilience to past struggles and to emerging challenges (14). As discussed in the first paragraph of this article, many Arctic communities have very high suicide rates, histories of colonization and oppression, and currently live with inequalities in housing, healthcare, education and employment (4, 6, 7, 9, 11, 22–27). Despite these challenges, historical trauma, and experiences of loss due to suicide of friends or family members, many youth have shown ingenuity, innovation and creativity in coping and a desire to be useful and appreciated by their families and communities (7, 9, 14). Far from being passive victims, youth in the Circumpolar North have demonstrated adaptability and active participation in advocating for themselves and dealing with their problems by using resources available to them and seeking guidance when needed to navigate their problems and difficulties (7).

This review finds no homogeneity among Indigenous communities in the Circumpolar North around communication or cultural norms for dealing with mental health issues. In Canadian Inuit culture, personal emotions are often kept private and aggression or confrontation is not usually how people express themselves (11, 63, 75). In contrast, in the Inupiaq (Alaskan Native group) culture, it is more common for people to be encouraged to share deep feelings with others in a public context (17). This points to the diversity between Circumpolar Indigenous groups and highlights the importance of ensuring that mental health programming and support is sensitive to and reflective of local culture and needs (*cf*. 76), and ensuring the participation of local healthcare providers, stakeholders and mental health experts in the creation of mental health resources (12).

Also connected to the need for contextually nuanced mental health programmes is the importance of developing mental health resources that are appropriate and relevant to youth and reflective of local perspectives of mental health and well-being (12, 23, 33, 63). In addition to bringing local values, perceptions and priorities to the forefront of mental health programming, it is equally important to provide the space and the opportunity for youth to bring their cultural values, skills and practices into their daily lives in ways that are meaningful for them and reflect their daily realities (7, 12, 14, 17, 19, 24, 41, 63).

## Conclusion

Current rapid rates of socio-cultural, political and economic change, combined with rapid rates of environmental change, underpinned by a history of colonization and current living conditions, creates a context of compounding stressors that increase youth susceptibility to mental health problems and contribute to rising negative health outcomes and disparities (5, 6, 8, 9, 12, 13, 19, 21). Indigenous youth in the Circumpolar North are influenced by these broad spatial and historical processes which shape their experience of and response to social, spiritual, physical, cultural, political, economic, and environmental stressors and transformations (6, 12, 13, 21, 26). Consequently, it is crucial to create the opportunities and environments where youth can successfully and positively navigate challenges and enhance and expand their resilience and adaptive capacities – all of which could help to foster healthy, thriving Circumpolar communities.

There is a need for future studies on Circumpolar Indigenous youth mental health to focus on resilience and protective factors to ensure that research advances in a positive manner and is reflective of local needs and understandings of mental health. The pathways and processes through which these factors protect is understudied and poorly understood and, as such, it is crucial that this research goes beyond identifying protective factors central to resilience and seeks to understand the pathways and processes through which these factors create and enhance individual and community resilience. This research will also need to be expanded to include the emergence of new technologies, social media platforms, socio-economic status, unemployment, housing, the quality of healthcare services and the education system. This additional information will assist communities and healthcare providers to enhance and expand understanding of how youth resilience and adaptive capacities to mental health challenges are developed and sustained. Enhancing this understanding may also help to further identify important resilience factors and to appreciate the interactions between and among protective factors.

It is imperative that these research results also be translated into action through culturally relevant community-level health and wellness programmes. For example, several authors from this review have created youth committees, youth action-groups, or regional youth councils that are fully involved in the research process, from setting objectives to facilitating research dissemination (3, 11, 16, 41). These opportunities provide youth with a chance to gain knowledge, skills and training that can help them problem solve and navigate challenges. Young people have the potential to become leaders within their respective communities and, to foster healthy communities and strong resilience, healthy and resilient youth must also be fostered and supported. This can be achieved with the creation of youth-focused mental health resources and programming that are reflective of local norms, values, customs and understandings of mental health, and that focus on enhancing and expanding the protective factors identified in this review through meaningful involvement of youth (3, 11, 16, 41).

## Footnotes

1 Ideally, a systematic literature review involves 2 or more people independently conducting the literature search and assessing the search results according to the pre-established criteria. Results are then compared to arrive at a final list of articles to review. One limitation of this study is that the search and assessment of articles was conducted by 1 person.

## Footnotes

2 For those articles that were empirical in nature, we reported only the overarching findings (i.e. what protective factors and/or causal mechanisms were identified) rather than specific numbers or measures as the articles that gave empirical results used varying measures that were difficult to compare and synthesize.
